# Supplementary material for: Cationic Lignocellulose Nanofibers from Agricultural Waste as High-Performing Adsorbents for the Removal of Dissolved and Colloidal Substances
Source: Polymers (Basel). 2022 Feb 24;14(5):910. doi: 10.3390/polym14050910 (PMC8912664; doi:10.3390/polym14050910)
Supplement: Supplementary file 1 [file polymers-14-00910-s001.zip › polymers-1602096-supplementary.pdf]

Supplementary Materials

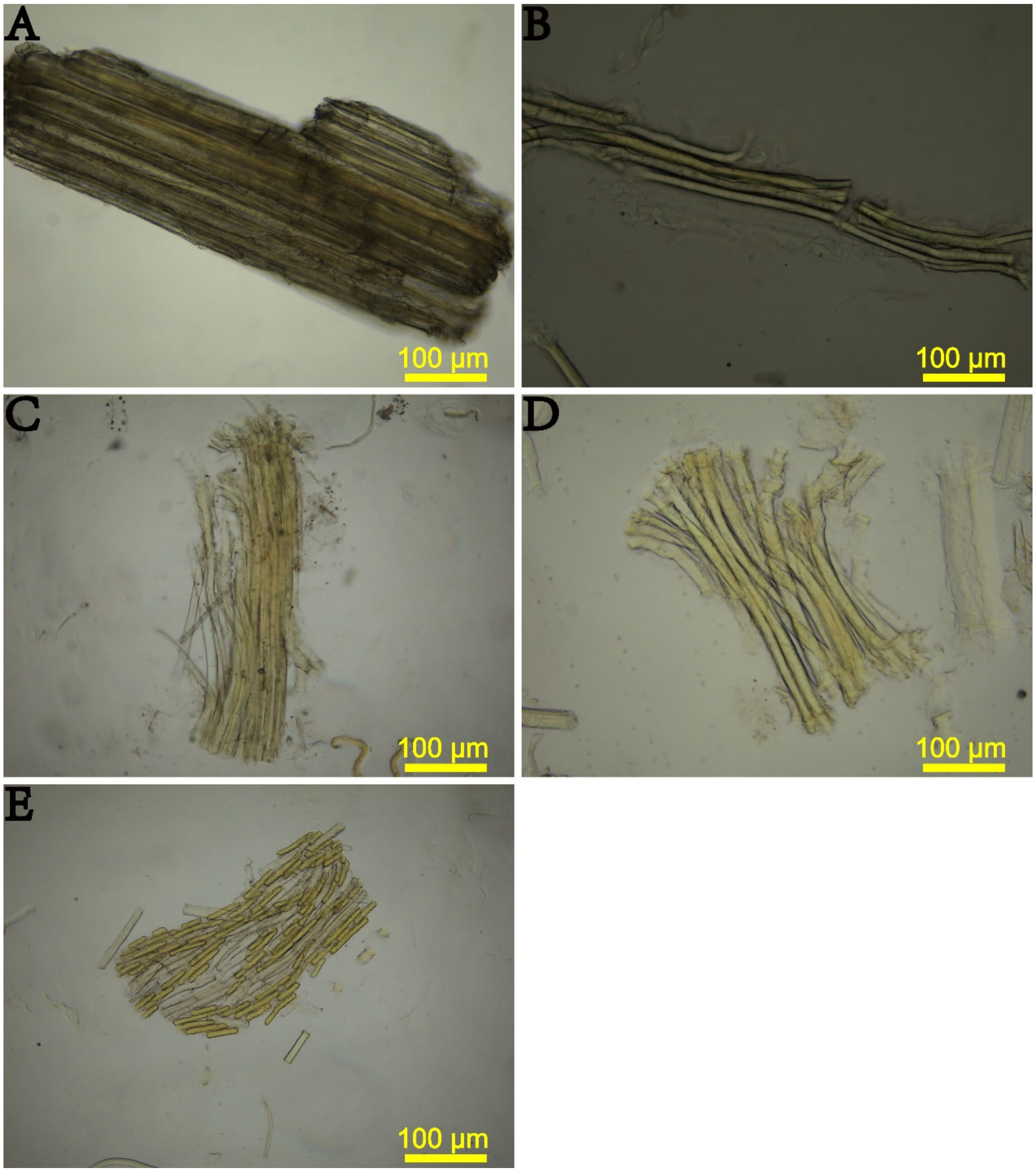

Figure S1. Optical microscope images of bagasse (A), the precursors of LCNF, CLCNF-1, CLCNF-2, and CLCNF-3 (B-E).

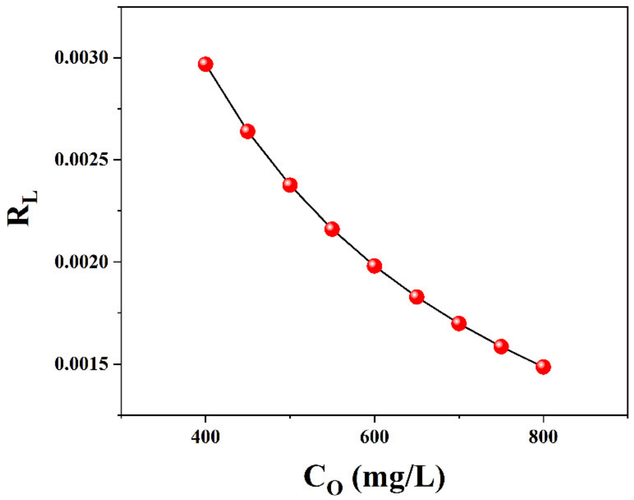

Figure S2. Separation factor of PGA adsorption by CLCNF-3.
